# Experiences of tobacco smoking and quitting in smokers with and without chronic obstructive pulmonary disease-a qualitative analysis

**DOI:** 10.1186/s12875-015-0382-y

**Published:** 2015-11-04

**Authors:** Eva A. M. van Eerd, Mette Bech Risør, Carolien R. van Rossem, Onno C. P. van Schayck, Daniel Kotz

**Affiliations:** Department of Family Medicine, Maastricht University Medical Centre, CAPHRI School for Public Health and Primary Care, P.O. Box 616, 6200 MD Maastricht, Netherlands; Department of Community Medicine, General Practice Research Unit, UiT The Arctic University of Tromsø, Tromsø, Norway; Institute of General Practice, Medical Faculty of the Heinrich-Heine-University Düsseldorf, Düsseldorf, Germany

## Abstract

**Background:**

Smokers with chronic obstructive pulmonary disease (COPD) seem to be a special subgroup of smokers that have a more urgent need to quit smoking but might find it more difficult to do so. This study aimed to explore which justifications for tobacco smoking and experiences of quitting were commonly shared in smokers with and without COPD, and which, if any, were specific to smokers with COPD.

**Methods:**

In ten primary healthcare centres in the Netherlands, we conducted semi-structured, in-depth interviews in 10 smokers with and 10 smokers without COPD.

**Results:**

Three themes were generated: ‘balancing the impact on health of smoking’, ‘challenging of autonomy by social interference’, ‘prerequisites for quitting’. All participants trivialized health consequences of smoking; those with COPD seemed to be less knowledgeable about smoking and health. Both groups of smokers found autonomy very important. Smokers with COPD were indignant about a perceived lack of empathy in their communication with doctors. Furthermore, smokers with COPD in particular had little faith in the efficacy of smoking cessation aids. Lastly, motivation for quitting was dominated by fluctuation and smokers with COPD specifically maintained that their vision of life was linked with quitting.

**Conclusions:**

The participants showed many similarities in their reasoning about smoking and quitting. The corresponding themes argue for a less paternalistic regime in the communication with smokers with attention required for the motivational stage and room made for smokers’ own views, and with clear information and education. Furthermore, addressing social interactions, health perceptions and moral agendas in the communication with smokers with COPD may help to make smoking cessation interventions more suitable for them.

**Electronic supplementary material:**

The online version of this article (doi:10.1186/s12875-015-0382-y) contains supplementary material, which is available to authorized users.

## Background

Chronic obstructive pulmonary disease (COPD) holds a leading position in morbidity and mortality worldwide [1]. In the Netherlands, approximately 320,000 people have COPD. Approximately 6000 people died in 2010 in the Netherlands with COPD as the primary cause [2].

Tobacco smoking is the most commonly encountered risk factor for COPD in developed countries [1, 3, 4]. An estimated 10 to 15 % of all smokers develop clinically significant airflow obstruction [5]. Furthermore, smokers with COPD have a higher mortality rate [1] and a more rapid decline in lung function than non-smokers with COPD [6]. Smoking cessation is the only evidence-based intervention which has been proven to slow down the accelerated decline in lung function in smokers with COPD [1]. Several evidence-based smoking cessation interventions are available but not yet widely used [7–10]. A systematic review has shown that smoking cessation support in smokers with COPD is best when a combination of psychological and pharmacological interventions is used [11].

The majority of smokers know that smoking is harmful to their health; and it is estimated that 50 % of smokers with COPD are amenable to smoking cessation support [12]. However, results from smoking cessation intervention studies comparing smokers with and without COPD have yielded ambiguous results. Higher, equal and lower smoking cessation rates in smokers with COPD were found compared to smokers without COPD [13–16]. Nevertheless, the prevalence of smoking in patients with COPD is still high and exceeds the rate of smoking in the general population [17]. A large population-based study showed that the prevalence of smoking currently was 35 % among patients with COPD compared with 22 % among patients without COPD [17]. This implies that when patients get a diagnosis of COPD many still continue smoking even though quitting is their best treatment option. Their chances of quitting might partly be reduced because of a higher level of tobacco addiction and susceptibility to develop depressive symptoms [18, 19]. Therefore, smokers with COPD seem to be a special subgroup of smokers that have a more urgent need to quit smoking, but might find it more difficult to do so [18].

A specific approach for smokers with COPD, while probably needed, does not yet exist. In order to create smoking cessation programs tailored to the needs of smokers with COPD, we need to gain their own views on smoking and quitting. Up until now, there have been some qualitative studies reporting perspectives of smokers with COPD [20–23]. Few of them have been able to identify concrete clinical implications. For example, Eklund et al. described how important it is to make sure that the smoker has the right intrinsic motivation before starting an attempt to quit [22]. In addition, Wilson et al. pointed out the importance of taking into account volatility in patients’ decision making and formulating realistic goals [21]. However, these studies only reported results of smokers with COPD, making it unclear whether these statements apply only to these smokers or are a reflection of smokers in general. As far as we are aware, there are only two studies comparing smokers with and without COPD [24, 25]. These showed a lot of similarities between the two groups [24, 25], but also some differences; smokers with COPD had higher tobacco consumption, higher nicotine dependence and a different pattern of smoking compared with smokers without COPD [24]. However, in these studies there is no qualitative data on smokers’ specific experiences regarding smoking cessation.

The aim of this qualitative study was to explore which justifications for tobacco smoking and experiences of quitting were commonly shared in smokers with and without COPD, and which, if any, were specific to smokers with COPD. Once researchers and healthcare providers understand the concerns of smokers with COPD regarding quitting and utilizing cessation treatments, they may address these problems in customised smoking cessation interventions in order to increase smoking cessation in smokers with COPD.

## Methods

### Setting and study population

Participants for the interviews were derived from a cross-sectional survey focusing on factors associated with smoking cessation in smokers with and without COPD [19]. Participants were recruited from the ‘Eindhoven Corporation of Primary Healthcare centres’ (SGE), which is a network of ten primary healthcare centres in the city of Eindhoven, the Netherlands, covering approximately 65,000 patients [26].

In January 2012, all patients from SGE with a recorded diagnosis of COPD (*N* = 1248) in their electronic medical record (EMR) and twice as many patients without COPD (*N* = 2466) received a questionnaire per postal mail and an enclosed form on which they could indicate if they were willing to participate in an interview. A total of 437 patients (31 %) with COPD and 875 patients (31 %) without COPD responded to the questionnaire. Among these, we identified 107 current smokers with COPD (24.5 % of 437) and 86 current smokers without COPD (9.8 % of 875). Thirty-four percent (36/107) of the patients with COPD and 53 % (46/86) of the patients without COPD met the inclusion criteria for attendance at the interview: willing to participate and being a current smoker with a smoking history of at least five years. We obtained written informed consent from all participants.

To provide elaborate understanding of smoking behaviour and smoking cessation, and supplement the cross-sectional survey, a purposive sample of 10 smokers with and 10 smokers without COPD was chosen by the coordinating researcher (EE) for interviewing. They were selected in order to include maximum variation in age and sex within both groups. Furthermore, age and sex, important risk factors for the development of COPD, were equally distributed between both groups.

### Data collection

Semi-structured in depth interviews of 60-90 min were conducted. An interview topic guide, consisting of four central topics, was developed, based on the literature and expertise in the research team [see Additional file 1] with the purpose of getting knowledge of the informants’ smoking narratives.

The interviews were performed in the participants’ homes to enable them to feel at ease and be open about their reflections on smoking. Furthermore, the interviewer attempted to adopt a non-judgmental attitude and bridle presumptions and prejudices to assure room for participants to express their thoughts freely on smoking and smoking cessation. The interviews were audio recorded. Furthermore, the interviewer (EE) wrote down non-verbal impressions and other notable information. Transcription was done by one researcher (EE).

Participants were given a €15 gift voucher after the interview was completed. The study was approved by the Medical Ethics Committee of Maastricht University, the Netherlands (approval number 11-4-145).

### Analysis

The topic of smoking and smoking cessation is, in most contexts, morally contentious. Attention must be paid to the questions posed by the interviewer and responses made by the interviewee, to avoid eliciting justifications or normative, ideal statements only. We believe that through a careful interview relationship we achieved data both consisting of justifications, explanations and patient-near experiences, reflecting what made sense to the participants as immediate concerns and thoughts. Still, in the analysis we paid attention to the moral context of the data and attempted not to infer what could not be justified.

Thematic analysis was used according to Braun and Clarke [27]. EE orthographically transcribed the interviews and imported them into NVivo 9 [27]. Data collection and analysis were carried out concurrently by one researcher (EE). EE checked the transcripts against the original audio recordings for accuracy. Next, EE identified initial codes and formulated these in English. A semantic approach was used which identified both the explicit and surface meaning units as well as reflected the smokers’ reality [27]. Primarily, we used an inductive thematic analysis, which means that EE coded the text without initially relating it to the aim of the study. The researchers, however, did have a certain interest in specific components regarding smoking cessation [27]. We realized a balance by giving some direction to the information-collection process (topic list) whilst also enabling different matters to evolve through the coding process. EE and DK checked the English codes back against the underlying Dutch quotes. Codes were then discussed in detail and sorted into categories and subcategories (EE and MBR). For example, there were categories titled ‘experiences of not being able to quit’ and ‘beliefs about the relationship between health and smoking’, with subcategories describing different experiences and beliefs the participants came up with. Next, the essential contents of the codes were interpreted into themes (EE and MBR). For example, the essence of all the codes describing thoughts about health and smoking resulted in the theme ‘balancing the impact on health of smoking’. EE checked if the resulting thematic map accurately reflected the meaning units evident in the dataset as a whole [27]. EE discussed the resulting thematic map with MBR and DK. Afterwards we checked if the themes and underlying codes contained differences between smokers with and without COPD.

Overall, we used a constant comparative approach to the data analysis with the aim to saturate the coded categories and themes. There was no need to extend the data collection to more than twenty participants, since the analysis made clear that the developing themes could be filled in and enriched from the data we had.

## Results

Characteristics of participants are displayed in Table 1. All smokers had started smoking at a time when this was socially acceptable. Most had started smoking because of social reasons.

**Table 1:** Characteristics of smokers with and without chronic obstructive pulmonary disease (COPD)

	With COPD (*n* = 10)	Without COPD (*n* = 10)
Female sex	40 (4)	40 (4)
Age, mean (SD)	60.0 (8.1)	62.6 (9.0)
Socioeconomic status [39]		
Low	22 (2)	0
Moderate	68 (6)	60 (6)
High	11 (1)	40 (4)
GOLD^a^ status (self-reported)		
Mild	33 (3)	n/a
Moderate	22 (2)	n/a
Severe	11 (1)	n/a
Very severe	0	n/a
I do not know	22 (2)	n/a
Level of nicotine addiction [40]		
Low	33 (3)	40 (4)
Medium	67 (6)	50 (5)
High	0	10 (1)
Motivation to quit^b^ [41], mean (SD)	2.4 (1.3)	3.2 (1.9)

Data are presented as % (n) unless stated otherwise. Discrepancies between total numbers and numbers of added subcategories are due to missing values

^a^GOLD = Global Initiative for Chronic Obstructive Lung Disease

^b^1-7 = highest level of motivation

### Theme 1: Balancing the impact on health of smoking

The participants weighted and interpreted facts on the relationship between smoking and health, and created their own judgements and justifications about the health risks of smoking. We noticed, for example, that some of them were balancing the health risks of smoking against the health risks of other addictive behaviours, such as eating too much.

…and if I can’t smoke then I’ll spend all day eating! What, then, is better for me? Because that way means you’ll end up far too fat. [interview 6 – without COPD]

Moreover, participants compared the impact on health of smoking with the impact on health of air pollution created by traffic and factories. They emphasized that air pollution is as bad or even worse for their health as smoking.

Living here on the Kennedylaan with the balcony doors open, drinking a cup of coffee in the morning sun – that’s much worse for you than smoking two packs of roll-up tobacco a day. Those cars that drive past and stop here for the traffic lights – now that’s real filth! [interview 4 – without COPD]

Examples such as these suggested that the participants did realize that smoking is bad for their health, because they compared it with something else which is unhealthy and stated that smoking is less bad. However, by talking about other people and other pollution mechanisms than their own smoking behaviour, they trivialized the health consequences of their own smoking behaviour. In other words, drawing on experiences and knowledge sources seemed to create a practical reasoning to justify their smoking behaviour.

Furthermore, the participants expressed inconsistent views about the causal relationship between smoking and disease. At the beginning of the interview, many participants in both groups said that they knew smoking is bad for their health. However, all through the interview, particular smokers with COPD would refine on their previous statement. Moreover, there were smokers with COPD who said they were unsure about the relation between smoking and having COPD.

…now if smoking really is the cause of that COPD, I am not sure; I don’t know. I have no real idea where it comes from. [interview 9 – with COPD]

Overall, participants did not differ much in their expressions on the causal relationship between smoking and disease. However, smokers with COPD did not express more awareness after having had the diagnosis and some smokers with COPD even remarked, possibly as justification or as fact, that the doctors had never told them smoking was bad for their respiratory health.

Finally, existential reflections played a part in reasoning about smoking and health. Smokers with COPD particularly expressed that how they envisioned life and death was interrelated with quitting.

…a while ago, I had a massive fear of death and that’s the time that I had a total change of heart. I don’t fear it at all anymore. That’s one of the reasons why I ask myself why should I go without that cigarette that I like so much? Why deprive myself? [interview 18 – with COPD]

Other smokers with COPD expressed similar feelings, reasoning that they did not mind dying, therefore giving up smoking was not relevant. One smoker with COPD argued that by continuing with smoking at least he knew what he would die of. As some of them had a particular fear of pining away [waste away in misery] if they were to quit smoking, they envisaged this not happening if they continued smoking. There was also one smoker without COPD who expressed such existential reflections. He had had an experience with a life-threatening disease, which made him realize that, in contrast with the smokers with COPD, he did not wish to die yet. In conclusion, smokers with COPD seemed to justify the fact that they were still smoking by existential reasoning; there was no need for them to quit because they did not mind dying from smoking. Conversely, the one smoker without COPD seemed interested to quit due to existential reasoning; he realized he did not want to die and this encouraged him to undertake a quit attempt.

### Theme 2: Challenging of autonomy by social interference

The impact of comments or advice from different sources, such as government, healthcare providers and family, seemed to interfere greatly with the smokers’ feelings and thoughts about smoking. Most comments in this regard had a negative weighting. The participants said that, in general people’s comments on smoking were experienced as being meddlesome. They frequently described feeling irritated, especially by ex-smokers’ comments.

Sometimes I reckon it’s exaggerated. Then I think, oh, come on, guys, please! Those who used to smoke the most have now stopped or are stopping, and they are the ones with the most to say when anyone else is smoking. [interview 2 – with COPD]

These feelings of irritation may be connected with a strong feeling of autonomy. All participants felt they needed freedom to decide to do as they wished. Furthermore, they felt they understood why general practitioners (GP) advised them to stop smoking. However, the participants mentioned in passing that they were irritated by smoking cessation advice, especially when it was very insistent: most of them would thus cut short conversations about smoking.

I just don’t say anything, I refuse to discuss the matter. It’s bad; I know that. I refuse to discuss it because you end up in an argument that leads nowhere: he [GP] makes all sorts of remarks such as, “It is really bad,” and then I say, “Yes, I know, but I am going to carry on doing it [smoking] anyway.” [interview 17 – without COPD]

Most of the participants had an opinion about continuing smoking and seemed not to be susceptible to external influences, such as advice from a GP. In addition, smokers did not feel like actively responding to the GP’s advice because they said they would sort it out themselves. Again, feelings of autonomy dominated. Furthermore, some smokers with COPD were indignant about the communication with specialists and GPs. This indignation seemed to be related to the amount and type of emotional attention given by the healthcare provider.

Personally, I think that the doctors of today are rather lax. You are left to scrabble after everything for yourself. There’s no real expression of sympathetic interest in retrospect on their part. [interview 9 – with COPD]

Smokers with COPD were indignant because they felt that, these days, healthcare providers did not show interest in them anymore. As one of the smokers with COPD justified: if doctors had more empathy for them, pieces of advice and comments on smoking and smoking cessation would stick in their memory better and would have a more encouraging effect. This might seem to be a justification only, but this also reflects the importance for doctors to build up solid relationships with their patients in order to gain trust. This trust is of particular importance for being able to discuss confidential and morally contentious topics, such as smoking. Finally, governmental regulations were not found to be motivating for quitting smoking according to all participants. Even though they had resigned themselves to the anti-smoking policies, they considered them to be ineffective, discriminating and out of proportion.

### Theme 3: Prerequisites for quitting

The overall experience with regard to ‘quitting’, was presented in rather negative terms; it was about not being able to quit. The participants often stated it was very difficult to quit and that they had little faith that they would be able to do so.

…and every time I decide to stop…for one reason or another, I just can’t manage…I really do want to and my wife does as well but that little bit of…will power…that is what’s lacking. [interview 3 – with COPD]

In addition, many smokers found a wide variety of preconditions that had to be fulfilled before they would be ready to make an attempt to quit. These preconditions, however, were susceptible to changes.

I did have something once…it was last year. It was my voice; it disappeared. Yes. Then I had to go to the Ear, Nose and Throat specialist and he told me that I had cancer of the vocal cords. And still I thought to myself, “Now, listen; if they were able to remove it then, then they can do the same next time.” So that’s why I started smoking again. [interview 9 – with COPD]

Furthermore, some of these conditions were not tangible, which made it difficult for outsiders to understand these conditions.

I say, “Yes, if a switch is flicked here [points at her head], then I’ll quit”. [interview 2 – with COPD]

A lot of participants reasoned that if they really wanted to quit, they could do it. They described this motivation as a feeling from inside. One of the participants illustrated this by saying ‘it’s all in the mind’. Many of these examples relate to intrinsic motivation to quit smoking. Most of the participants had changeable explanations and justifications concerning their willingness to quit. They seemed to lack intrinsic motivation and had difficulties obtaining it. However, they could give examples of extrinsic motivators such as health education or comments from significant others, but these were mostly only potential motivators, and might interfere with feelings of autonomy (theme 2). We did not find examples showing that in the end they actually persuaded them to quit. In addition, some motivators, such as health complaints, would disappear over time and therefore not stimulate long-term abstinence. When these health problems faded away, smoking again would easily start.

Furthermore, participants often said that life-events interfered with their motivation to quit and with the maintenance of an attempt to quit. Life-events would prevent them from starting an attempt to quit and also from maintaining the effort.

Finally, the availability of various smoking cessation aids was not motivating for quitting either and smokers with COPD in particular expressed that using smoking cessation aids would only make sense and be effective if one were ‘ready’ to quit. Nicotine replacement therapies were not found to be effective by all participants because, afterwards, it was claimed that they did not work or had a dirty taste or, beforehand, it was thought that they did not stop the craving which was seen to be the main difficulty in smoking cessation. The participants reported diverse views on medication, such as bupropion and varenicline. However, smokers with COPD were more negative about these medications. Finally, all participants were sceptical about the use of e-cigarettes. Some stated that by using them it made them smoke more and they would not solve the craving problem. Those who were somewhat positive about e-cigarettes did not use them as an aid to quit but as a temporary alternative. In general, smokers wanted aids to solve their craving to smoke and fully to replace the effect of a cigarette.

## Discussion

All participants reported a lot of shared justifications concerning smoking and experiences of quitting. They all trivialized the health consequences of smoking and also felt a threat of social interference in their autonomy which did not motivate them to quit. However, smokers with COPD seemed less knowledgeable about the relationship between their health and smoking; were indignant about a perceived lack of empathy from doctors; expressed in particular that their view of life was interrelated with quitting; and had less faith in the efficacy of smoking cessation medications.

It became apparent from the interviews that smokers generally trivialized the health consequences of smoking. However, most of the smokers did make comments that showed at least some consciousness of this relationship. It is possible that answers on this topic from smokers in general, and smokers with COPD in particular, reflect a construction of justification of smoking as a certain style of reasoning. However, we also argue that this is also what made sense to their situation and lived experience of being a smoker, the performance and embodiment of a distinct health and risk discourse. Another aspect is that answers on this topic in general might be biased by optimism in order to reduce the psychological need to quit smoking. This cognitive dissonance could be a coping mechanism to manage their lifestyle [28]. In general, trivializing, comparing and, in that way, balancing different knowledge sources are ingredients often involved in creating and justifying a certain lifestyle [29, 30].

Autonomy was another key issue for smokers. It seemed that advice and comments were mostly experienced as being meddlesome, even when well-intended. This made it challenging for healthcare providers to win acceptance to discuss smoking and quitting. In the Netherlands, individualism is highly valued, as in other western European countries where qualitative research on smokers with COPD has found that these smokers had a great need for autonomy, and decisions had to be taken independently in order to retain that autonomy [22]. In that study, smokers with COPD sympathized with the idea of receiving help and support, but without being patronized [22]. The same need for supportive emotional communication was seen in our smokers with COPD, who found that doctors lacked empathy for, and interest in, their patients.

We found that motivation for quitting was volatile and sometimes intangible, both in smokers with and without COPD. In several behavioural change models, motivation is one of the key elements to explain changes in health behaviour [31–33]. In previous research, smokers with COPD have acknowledged the need for intrinsic motivation and will power to stop smoking, but they were unsure about how to obtain and maintain will power [21]. Similarly, in our study, motivation for quitting was often referred to as ‘something from inside’ which was not tangible and smokers were unsure how to achieve it. Nevertheless, smokers described intrinsic motivation as the most important motivator for successful quitting. In addition, extrinsic motivation can stimulate quitting as a result of an external source [21, 34]. In our study, smokers did not seem to believe in the efficacy of extrinsic motivators. Similar to the findings of previous research, external sources were found to be demotivating at times; life events often provided reasons for never finding the time to focus on smoking cessation [22]. In conclusion, motivating and demotivating factors were volatile, which was also seen in previous research on smokers with COPD [21].

Most smokers thought of smoking cessation aids as being ineffective. Smokers with COPD in particular stated that using smoking cessation aids would only make sense and be effective if one were ‘ready’ to quit. With these expressions they seemed to have little faith in the efficacy of smoking cessation aids. A recent study in the Netherlands showed that a low level of knowledge about the evidence-base of aids was possibly due to the promotion of ineffective commercial cessation methods [35]. Furthermore, the lower socio-economic status in the group of smokers with COPD might have been negatively associated with their level of faith in smoking cessation aids, as they are likely to be less educated, hence more likely to dismiss or misunderstand the evidence for smoking cessation aids. In addition, one could also discuss the perception of when a treatment is effective: smokers in our study often expressed that smoking cessation aids were ineffective because they did not make them quit smoking after their use. Smokers with COPD in particular were negative about the side effects and inefficacy of medication such as bupropion and varenicline, both before and after they had used them. This attitude might be counterproductive because smokers with COPD are more nicotine-dependent [19, 24], which makes this evidence-based medication of particular importance to this group of smokers [36, 37].

The study has strengths: the background and interview training of the interviewer (EE) increased the quality of the interviews. Participants were interviewed in their own home for comfort which contributed to open conversations about a confidential and morally contentious topic. During the interview EE tried not to be guided by the knowledge of whether an interviewee was a smoker with or without COPD and also coded both groups as a whole in order to prevent guidance by potential differences. EE wrote down non-verbal impressions during the interview and transcription: in this way, an analysis of both verbal statements, non-verbal impressions and contextual conditions for data production was realized. In addition, researcher triangulation was used in order to overcome intrinsic bias.

Some limitations also need to be mentioned: selection bias might have occurred as the participants engaged in the interviews could have held stronger views about smoking and quitting than the ones that were not interested to participate. However, we still saw a diversity of characteristics and opinions coming up, which may indicate that the results reflect the views of the general population of smokers. A pragmatic number of interviews was done initially, however we found that the analytic process made our arguments coherent, i.e. we decided that no further interviews were necessary. The generation of the initial codes was done by one researcher (EE) only and MBR, who helped initiate the codebook, was not able to read the interviews herself because of a language mismatch. However, considerable time was spent on discussion and explanation of the contents of the interviews. The study’s generalizability may be limited but is determined by its overall design. However, we believe a thorough and consistent design as well as analysis gives credibility to analytic generalisation, i.e. the development of valid, rich findings feeding into a general understanding of a field of research which we hope to have presented [38].

## Conclusions

The participants showed many similarities in their reasoning about smoking and quitting. Both groups trivialized the health consequences of smoking and had no faith in the efficacy of smoking cessation aids. Furthermore, all participants reported fluctuation in motivation to quit and the importance of autonomy in making their own decisions. With regards to the smokers with COPD, some specific remarks should be made: they seemed less knowledgeable regarding the relation between their health and smoking and had less faith in the efficacy of medication. Besides, they expressed more existential reflections on their smoking behaviour and were indignant about the perceived lack of empathy from doctors.

For future smoking cessation interventions the corresponding themes argue for a less paternalistic regime when communicating with smokers. Smoker’s views need to be explored, particularly regarding their motivation to quit. Clear information and education needs to be provided on the efficacy of smoking cessation aids and the negative consequences of smoking on health. In addition to these general recommendations for smoking cessation interventions, some recommendations for COPD tailored smoking cessation interventions can be made: GPs should, in their communication with smokers with COPD, take more time to explore social interactions, health perceptions and moral agendas that influence thoughts, feelings and what makes sense concerning smoking and quitting. Once the healthcare provider has gained a common understanding and trust, information and education about the relationship between COPD and smoking, and the use and efficacy of various smoking cessation aids, will possibly be appreciated more and absorbed better by the smokers with COPD.

## Additional file

Additional file 1:
**Interview topic guide.** (DOCX 17 kb)

## Abbreviations

COPD: Chronic obstructive pulmonary disease
EMR: Electronic medical record
GOLD: Global initiative for chronic obstructive lung disease
GP: General practitioner
SGE: Eindhoven corporation of primary healthcare centres
