# Supplementary material for: Experiences of tobacco smoking and quitting in smokers with and without chronic obstructive pulmonary disease-a qualitative analysis
Source: BMC Fam Pract. 2015 Nov 4;16:164. doi: 10.1186/s12875-015-0382-y (PMC4634152; doi:10.1186/s12875-015-0382-y)
Supplement: Additional file 1: — Interview topic guide. (DOCX 17 kb) [file 12875_2015_382_MOESM1_ESM.docx]

**Interview guide**

***1. the rationale of smoking and its significance in everyday life***

Can you tell me about your smoking habits in general?

How did you start smoking? (when? influencing factors?)

How did it develop? (smoking habit)

What does smoking mean to you? (why do you smoke?)

When do you smoke? (specific situations)

Where do you smoke? (specific room)

***2. the influence of the environment on smoking and smoking cessation***

Can you tell me about the influence of your environment on your smoking habits?

Are there any persons in your family who influence your smoking habits?

How/when are they influencing your smoking habits?

Are there any other persons in your environment who influence your smoking habits?

How/when are they influencing your smoking habits? (legislation?)

Are there any health-care professionals in your environment that influence your smoking habits?

How/when are they influencing your smoking habits?

How do your smoking habits influence your interaction with other people, your daily contacts? (social isolation?)

***3. emotional dimensions of being a smoker***

Can you tell me about the emotional aspects of being a smoker?

When do you feel good about smoking? Why do you feel good about smoking then?/What do you feel? (self-esteem? motivations?)

When do you feel bad about smoking? Why do you feel bad about smoking then?/What do you feel? (self-blame, guilt, shame, personal weakness, psychological distress)

How do you feel when you are asked about smoking? (judged? do you feel able to be honest about your smoking?)

How do you believe other people think about smoking? Why do you believe that?

Do you think people blame you when you are ill? Do you blame yourself?

How do you feel when you attend the health-care appointments? (do you feel that you can be honest about your smoking habits? do you feel health care providers understand you? do you feel able to approach your doctor for help to give up smoking?)

How do you respond to advice about smoking and your smoking habits? (avoid confrontation?) (patronizing?)

***4. current barriers and potential facilitators of making a quit attempt and using primary care smoking cessation treatments***

Now I would like to talk about quitting and quit attempts

Have you ever tried to quit?/Have you ever thought about quitting? -> IF YES, keep your last attempt in mind while answering the next questions (if possible compare with other previous quit attempts):

What triggers you to make a quit attempt?/What triggers you to think about a quit attempt? (circumstances, experiences, knowledge, money)

How does a quit attempt make you feel?/How does thinking about a quit attempt make you feel?

How does it feel when the quit attempt does not succeed?

What holds you back from making a quit attempt? (circumstances, experiences, knowledge, money, fill the void)

What information did you receive about the benefits of quitting? From whom? (primary care?)

Are any persons more legitimate to you to help you quit? Significant others? (primary care?)

What information did you receive about the side-effects of quitting? (primary care?)

What treatments have you tried, if any? (pharmaceutical, behavioural)

What do you think about these treatments; (intensive) counselling, NRT/bupropion/varenicline? (even if you did not try them)

How do you think about smoking less as an alternative for complete cessation?

How do you think about slow reduction to quit smoking as an alternative for abrupt quitting?

Is there anything healthcare could do to help you attempt to change your smoking habit? (counselling/treatment?)
